# Statistical Inference for Ergodic Algorithmic Model (EAM), Applied to Hydrophobic Hydration Processes

**DOI:** 10.3390/e23060700

**Published:** 2021-06-01

**Authors:** Emilia Fisicaro, Carlotta Compari, Antonio Braibanti

**Affiliations:** Food and Drug Department, University of Parma, 43121 Parma PR, Italy; emilia.fisicaro@unipr.it (E.F.); carlotta.compari@unipr.it (C.C.)

**Keywords:** hydrophobic hydration process, ergodic algorithmic model (EAM), thermal equivalent dilution (TED), binding potential functions, intensity entropy, density entropy

## Abstract

The thermodynamic properties of hydrophobic hydration processes can be represented in probability space by a Dual-Structure Partition Function {*DS-PF*} = {M*-PF*} · {*T-PF*}, which is the product of a Motive Partition Function {M*-PF*} multiplied by a Thermal Partition Function {*T-PF*}. By development of {*DS-PF*}, parabolic binding potential functions α) *R*ln*K_dual_* = (−Δ*G°_dual_/T*) ={*f(*1/*T*)*g(T)} and β) *RT*ln*K_dual_* = (−Δ*G°_dual_*) = {*f(T*)*g(*lnT*)} have been calculated. The resulting binding functions are “*convoluted*” functions dependent on the reciprocal interactions between the primary function *f(*1/*T*) or *f(T*) with the secondary function *g(T*) or *g*(*lnT*), respectively. The binding potential functions carry the essential thermodynamic information elements of each system. The analysis of the binding potential functions experimentally determined at different temperatures by means of the Thermal Equivalent Dilution (TED) principle has made possible the evaluation, for each compound, of the *pseudo*-stoichiometric coefficient ±*ξ_w_*, from the curvature of the binding potential functions. The positive value indicates convex binding functions (Class A), whereas the negative value indicates concave binding function (Class B). All the information elements concern sets of compounds that are very different from one set to another, in molecular dimension, in chemical function, and in aggregation state. Notwithstanding the differences between, surprising equal unitary values of *niche* (cavity) formation in Class A <Δ*h_for_*>_A_ = −22.7 ± 0.7 kJ·mol^−1^ *·ξ_w_*^−1^ sets with standard deviation σ = ±3.1% and <Δ*s_for_*>_A_ = −445 ± 3J·K^−1^·mol^−1^·*ξ_w_*^−1^J·K^−1^·mol^−1^·*ξ_w_*^−1^ with standard deviation σ = ±0.7%. Other surprising similarities have been found, demonstrating that all the data analyzed belong to the same normal statistical population. The Ergodic Algorithmic Model (EAM) has been applied to the analysis of important classes of reactions, such as thermal and chemical denaturation, denaturation of proteins, iceberg formation or reduction, hydrophobic bonding, and null thermal free energy. The statistical analysis of errors has shown that EAM has a general validity, well beyond the limits of our experiments. Specifically, the properties of hydrophobic hydration processes as biphasic systems generating convoluted binding potential functions, with water as the implicit solvent, hold for all biochemical and biological solutions, on the ground that they also are necessarily diluted solutions, statistically validated.

## 1. Introduction

The thermodynamic properties of hydrophobic hydration processes in water have been analyzed by us in a set of articles [1,2,3,4]. The thermodynamic properties of these systems are described by setting a Dual-Structure Partition Function {*DS-PF*} = {M*-PF*} · {*T-PF*}, as product of a Motive Partition Function {M*-PF*} multiplied by a Thermal Partition Function {*T-PF*}. This means that every hydrophobic hydration system in water is biphasic, constituted by diluted solution and by water, as implicit solvent at constant potential *μ_s_*. By development of {*DS-PF*}, the binding potential functions α) *R*ln*K_dual_* = (−Δ*G°_dual_/T*) = {*f(*1/*T*)*g(T)} and β) *RT*ln*K_dual_* = (−Δ*G°_dual_*) = {*f(T*)*g(*lnT*)} have been proved to hold for every hydrophobic process. The binding potential functions are *convoluted* functions, indicating as *convolution* the reciprocal interactions of the primary function *f*(1/*T*) or *f*(*T*) with the secondary function *g(T*) or *g*(*lnT*), respectively. The extrapolatio ns of the thermodynamic functions *f*(1/*T*) or *f*(*T*) for each compound Δ*H_dual_* to *T* = 0 and Δ*S_dual_* to ln*T* = 0, respectively, are allowed because the coefficient Δ*C_p,hydr_* has been demonstrated to be constant and independent from the temperature for both mathematical and chemical constraints [5]. The extrapolations are correct because, for experimental data taken above 273 K, the inferior integration limits to *T =* 0 *K* and to ln*T* = 0, respectively, are equivalent because the interval (0 → 273 or more) is practically equal to the interval (1 → 273 or more).

The binding potential functions have been determined for many different systems and processes, for example for the solubility in water of noble gases and for protein denaturation, for protonation of carboxylic acids and for micelle formation [1,2 and reference therein]. Notwithstanding the evident structural, functional, and dimensional differences, every binding function has been found to be dependent on the same coefficients. At the end of the calculations, we have found that beyond significant information, extracted from the terms extrapolated to *ξ_w_* = 0, the unitary (unitary means for *ξ_w_* = 1) binding functions presented important similarities and analogies. Every function depends on the heat capacity of the system Δ*C_p,hydr_* and on a stoichiometric coefficient *ξ_w_*. The latter coefficient measures the number of water molecules W_III_ involved. Then, we have chosen to pass to an analysis of the general statistical validity of the coefficient found in different compounds and in different classes of reactions.

## 2. Results

### Statistical Inference: User-Friendly Functions

The extrapolation of the thermodynamic functions for each compound Δ*H_dual_* to *T* = 0 and Δ*S_dual_* to ln*T* = 0, respectively has made possible the disaggregation of the thermodynamic functions Δ*H_dual_* and Δ*S_dual_* in two parts, motive (or work) and thermal (or compensative), as proposed by Lumry [6] since 1980. The disaggregation in motive and thermal functions has been done successfully for all the experimental data in the literature concerning hydrophobic hydration process. The analysis of the thermal components by means of Thermal Equivalent Dilution (TED) [7] has made possible the evaluation of the *pseudo*-stoichiometric coefficient ±*ξ_w_* in each compound, from the curvature of the convoluted binding potential functions [1,2,3,4].

The separate motive functions, instead, can be analyzed and disaggregated by considering groups of compounds, i.e., for all the compounds in one family of reactions, by taking advantage of the previously determined coefficient *ξ_w_* of each compound. For instance, we have considered together, either obtained by us either by other researchers all the values of Δ*H_mot_* and Δ*S_mot_* for all non-polar gases, then for all liquids, then for all carboxylic acids, then for all micelles having analogous structures with increasing length of the chain, then for all proteins belonging to a homogeneous group such as the family of lysozymes, etc. For each family, we have plotted Δ*H_mot_* and Δ*S_mot_* of each component of the family against the respective number ξ*_w_*, previously determined by applying TED [5]. The disaggregation of enthalpy and entropy motive functions for non–polar gases are reported in Table 1.

We want to stress the point that the difference between one another group of compounds in structure, in molecular size, in aggregation state is remarkable. We call the attention, for example, on the differences existing between the determination of the solubility in water of non-polar gases and the denaturation of a protein: the molecular size of a gas is extremely different from that of a macromolecule, and the aggregation state as well. Moreover, radically different experimental methods have been employed in the various processes. Nonetheless, the motive functions of all the groups, when plotted as the functions of *ξ_w_*, have given in any case significant linear diagrams with the same unitary slope [1,2]. 

For the groups where the heat capacity Δ*C_p,hydr_* was positive (Class A) the analysis has yielded the following expressions—for enthalpy:

Δ*H_mot_* = Δ*H*_0_^(^*^ξw^*^ = 0)^ + *ξ_w_*Δ*h_for_* (1)

and for entropy:

Δ*S_mot_* = Δ*S*_0_^(^*^ξw^*^ = 0)^ + *ξ_w_* Δ*s_for_* (2)

In the families having negative heat capacity Δ*C_p,hydr_* < 0 (Class B), the expressions were found to be for enthalpy:

Δ*H_mot_* = Δ*H*_0_^(^*^ξw^*^ = 0)^ + *ξ_w_* Δ*h_red_* (3)

and for entropy:

Δ*S_mot_* = Δ*S*_0_^(^*^ξw^*^ = 0)^ + *ξ_w_* Δ*s_red_* (4)

The unitary functions calculated by disaggregation of the motive thermodynamic functions of every hydrophobic hydration process can be represented by a paradigmatic scheme (Table 2) composed by three terms, whereby each term can be associated to a specific reaction step. The single terms in the paradigm of Table 2 can be calculated.

The motive enthalpies in each family of homologous compounds and the unitary functions calculated by disaggregation of the motive entropies in each family are reported for Class A and for Class B in Table 3.

The unitary functions Δ*h_for_* and Δ*s_for_* in Class A and Δ*h_red_* and Δ*s_re_*_d_ in Class B, respectively, show important similarities and analogies. The mean values of the unitary functions in the two Classes A and B are reported in Table 4. The values of each unitary function present a small variability around the mean in both Classes. The same homogeneity of results was found in both Classes. The residual error is within the limits of a possible experimental error, thus validating statistically the model. Moreover, we have found that the unitary values of Class B are equal to the corresponding values of Class A, with sign reversed (Table 4). 

The statistical self-consistency of unitary data obtained by extremely different systems represents a decisive validation of the Ergodic Algorithmic Model (EAM). 

From a thorough analysis of the data in Table 4, *a* one can remark the large value of the negative unitary entropy change in Class A, <Δ*s_for_*>_A_ = −445 ± 3 J·K^−1^·mol^−1^·*ξ_w_*^−1^ and the corresponding unitary positive entropy change <Δ*s_red_*>_B_ = +432 ± 4 J·K^−1^·mol^−1^·*ξ_w_*^−1^ in Class B.

The fact that the values of the unitary thermodynamic functions in Class B are the same as the corresponding values in Class A with opposite sign confirms the hypothesis that in Class A and in Class B we are dealing with the same unitary processes but in opposite directions in the two Classes: reaction **A**(*ξ_w_*W_I_ → *ξ_w_*W_II_ (iceberg + *ξ_w_*W_III_) with phase transition in Class A and reaction **B**(−*ξ_w_*W_III_– *ξ_w_*W_II_ (iceberg → *ξ_w_*W_I_), in Class B with opposite phase transition. The large value of the negative unitary entropy change in Class A can be assumed to be a result of iceberg formation in connection with the dissociation [5] of +*ξ_w_* water molecules W_III_. By the name of “iceberg” we intend the complex between the hydrophobic molecule and the water W_II_, surrounding it, as a whole. In contrast, the large value of the positive unitary entropy change in Class B is in accordance with the association [5] of –*ξ_w_* water molecules W_III_ with iceberg reduction. 

The iceberg formation in Class A is equivalent to reducing the volume of the solvent (Δ*V_solvent_* < 0 = −*V_cav_*, with *V_cav_* = +*ξ_w_V*_WI_) and to increasing the concentration of the solute with configuration entropy loss, whereas in Class B the positive value of the unitary entropy change indicates that there is a process of iceberg reduction. Iceberg reduction means an increase of solvent volume (Δ*V_solvent_* > 0 = −*V_cav_*, with *V_cav_* = −ξ*_w_V*_W__I_) with dilution of the solute and consequent configuration entropy gain. The enthalpy-change for iceberg formation (Class A) is negative (<Δ*h_for_*>_A_ = −22.7 ± 0.7 kJ·mol^−1^·*ξ_w_*^−1^) thus indicating that clustering interaction with hydrophobic molecules and water (W_II_) is favored with respect to the clustering interaction H_2_O (W_I_) -- H_2_O (W_I_). We note that W_II_ is part of the solute and the preferred reaction is of concern of the Motive Partition Function {*M-PF*}.

The favorable enthalpy change is contrasted by the large negative entropy change due to concentration of the molecule, again an element of {*M-PF*}. The low solubility of gas re-establishes the free energy balance. On the other hand, in the process of Class B for iceberg reduction the enthalpy change is unfavorable but the entropy change is largely positive (<Δ*s_red_*>_B_ = +432 ± 4 J·K^−1^·mol^−1^·*ξ_w_*^−1^). We observe that in processes of Class B, as for example in micelle formation, the need of a balance by low solubility has decayed and the micelles are soluble. 

The preference by a system for a process of Class B (hydrophobic hydration) rather than that of Class A (hydrophilic hydration) is determined by the favorable large entropic unitary functions.

The finding that it is the entropy change to lead the reactions disproves the theory, as described by Ben Naim [8], that hydration might be determined by solute-solvent energetic interactions that should be either stronger (attractive) or weaker (repulsive) than the corresponding solvent–solvent interaction in the system, giving rise to hydrophobic or hydrophilic hydration, respectively.

We can calculate the concentration change, corresponding to the unitary entropy change. We recall (see Table 3) that the mean value for Classes A and B is <Δ*s_r_*>_A,B_ = 438.5 ± 6.5 J·K^−1^·mol^−1^. From Δ*S*_X_/*R* = −ln[X] (438.5/8.3145 = 52.74236) we calculate the unitary number of molecules *N*_Un_ = 0.802304·10^23^. Hence, by dividing by the Avogadro number *N*_Avo_ = 6.02214·10^23^, we can calculate the molecular (and molar) fraction *x* = 0.802304/6.02214 = 0.13322575 (13.32%).

It is important to point out that the cavity as calculated by statistical thermodynamic methods (e.g., Potential Distribution Theorem (PDT) [9]) is inconsistent with iceberg formation or reduction considered in this article. The former statistical cavity concerns exclusively the non-reacting ensemble of the solvent (*NoremE*), involving thermal functions without any effect on free energy and on thermodynamic potential *μ_s_*. The Potential Distribution of the Theorem (PDT) is not-existent because the solvent (as Implicit Solvent) in hydrophobic hydration processes and in biochemical reactions is at constant potential *μ_s_*: the Thermal Partition Function {*T-PF*} cannot give any contribution to free energy and to potential *μ_s_.* In contrast*,* the iceberg formation (or iceberg reduction) considered in this article concerns the motive functions with formation (or reduction) of iceberg by transforming water W_I_ into W_II_ and W_III_, or vice-versa. This process of iceberg formation, in Class A, reduces the volume of the solvent W_I_. In such a way, it modifies the thermodynamic state of the solute (by changing dilution and, therefore, configuration density entropy), which is of concern for motive functions (Δ*G_mot_ ≠* 0). Water W_II_, in fact, becomes part of the molecule of the solute (as iceberg sheath) and enters as component of the equilibrium constant in the solute motive partition function {*M-PF*}. In Class B, the opposite process takes place, with expansion of the solvent W_I_ thus modifying the thermodynamic state of the solute. The transformations in the thermodynamic state of the solute, including W_II_, are, again, of concern for motive partition functions {*M-PF*} and, consequently, for motive free energy.

The Ergodic Algorithmic Model (EAM) offers a complete picture of every hydrophobic hydration process, considering the steps of:

(i) Subdividing the apparent partition function into thermal {*T-PF*} and motive {*M-PF*} partition functions;
(ii) Determining iceberg formation or iceberg reduction in a niche within the field of motive partition function;
(iii) Considering two types of water clusters W_I_ and W_II_, together with free molecules W_III_;
(iv) Determining the number ±*ξ_w_* of water clusters W_I_ (phase change) from curvatures of the binding potential functions α) *R*ln*K_dual_* = {*f*(1/*T*)*g(*T*)} and β) *RT*ln*K_dual_* = {*f*(*T*)*g(ln*T*)}, calculated from sets of equilibrium constants measured at different temperatures and treated by thermal equivalent dilution principle;
(v) assigning to W_I_ the role of solvent (implicit solvent);
(vi) attributing any change of configuration entropy of W_II_ and W_III_ to the motive partition function {*M-PF*} (solute) and not to the thermal partition function {*T-PF*} of implicit solvent.

Altogether these elements concur to describe in detail the behavior of many biochemical reactions, so important for the description of biological processes. In this regard, we cannot forget the criticism by Lumry [6], who considered that the thermodynamic functions applied to biochemical equilibria were not user-friendly. He thought that the causes of the unreliability of these thermodynamic functions were that nobody used to subdivide the thermodynamic functions into thermal and work components, and nobody had considered the dual nature of hydrophobic solutions. These statements by Lumry represent a splendid insight into the problem of hydrophobic hydration. By applying the two conditions foreseen by Lumry, we have been able to calculate reliable unitary functions <Δ*h_for_*>_A_, <Δ*s_for_*>_A_, <Δ*h_red_*>_B_, and <Δ*s_red_*>_B_. The statistical analysis of the unitary data in Table 4 not only demonstrates that Δ*C_p,hydr_*, is constant but also that we can identify in the unitary functions the *user-friendly functions* hoped for by Lumry [10]. The statistical analysis has been extended to a significant number of different compounds. By employing the unitary functions of Table 4, if we can define previously, by applying TED, to sets of equilibrium constants measured at different temperature, the number ±*ξ*_w_ of molecules W_III_ involved, we can calculate the motive thermodynamic functions for iceberg formation or reduction in any new hydrophobic hydration process.

## 3. Discussion

### 3.1. Water in Thermal and Chemical Denaturation

**Figure 1:**
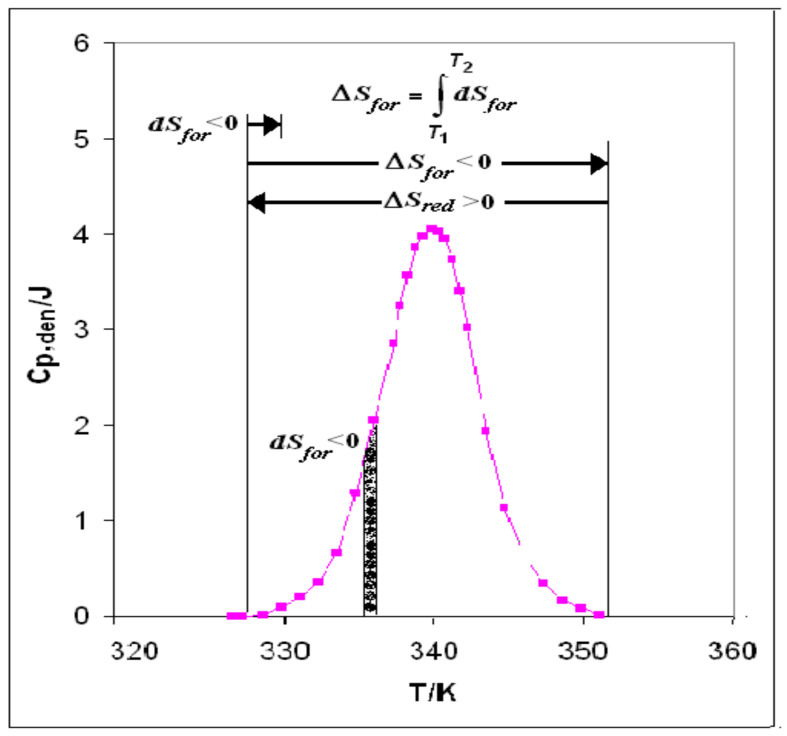
Thermal denaturation. Heat supply creates iceberg with negative entropy production. The integral entropy Δ*S_for_* at denaturation compensates exactly the integral folding entropy Δ*S_red_* (Δ*S_red_* + Δ*S_for_* = 0).

The statistical analysis has validated the unitary values found in different compounds. In the following paragraphs we want to analyze the specific reactions that were experimentally found to solve many questions so far not yet explained in the literature. We start by analyzing the displacement of the equilibrium between the different forms of water, to explain any transformation of the macromolecules, particularly in denaturation processes. A reasonable explanation of the mechanism of thermal denaturation is that the folded native protein had been formed through a process of hydrophobic association analogous to that of micelle formation, with an outstanding positive entropic contribution. We recall that the hydrophobic bonding is driven by the positive entropy change, ΔS_red_ > 0 produced as the consequence of the condensation of ξ_w_ water molecules (W_II_ + W_III_) into water W_I_, with iceberg reduction **B**(−ξ_w_W_III_ − ξ_w_W_II_ (iceberg) → ξ_w_W_I_. To the folded native protein can be assigned unitary values of the thermodynamic stepwise functions equal to those of the denaturation steps, with sign reversed. In the opposite reaction **A**(ξ_w_W_I_ → ξ_w_W_II_ (iceberg) + ξ_w_W_III_), taking place at denaturation, W_I_ is that part of the system that is giving rise to a change of phase, from structured to fluid state. When the heat supply starts, the heat moves a cluster from the solvent W_I_ thus generating one cluster W_II_ and a molecule of water W_III_, thus displacing the equilibrium toward the fluid state and formation of iceberg. The whole process takes place through three steps (Figure 1):

(1) *Start*: The heat supplied to the system generates melting of some clusters of water W_I_ to give W_II_ +W_III_, creating the niche wherein iceberg is formed. In fact, the creation of the niche with iceberg reduces the solvent (W_I_) volume and produces negative entropy (d*S_for_* < 0), thus beginning to cancel the positive entropic contribution of protein folding (Δ*S_red_* + d*S_for_*).
(2) *Scanning*: The process of heat supply continues until the integral entropy:
  *_T_*_2_ *ΔS_for_* = ∫ d*S_for_* *^T^*^1^ (5)
  cancels completely the positive entropy of folding and causes disruption of the hydrophobic bonds, at least those around the active site, that had been keeping the native protein folded.
(3) *Final:* At this stage (Δ*S_red_* + Δ*S_for_* = 0), the whole positive entropy contribution produced by folding is cancelled: the disruption of every hydrophobic bond is completed, and the denatured state has become the stable one. The denaturation process consists in the disruption, through iceberg creation and negative entropy production, of the hydrophobic bonds that had been keeping the chains folded in the native protein.

**Figure 2:**
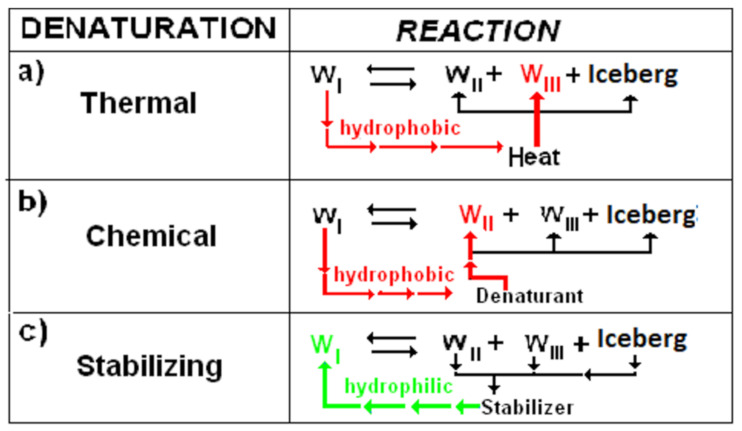
Types of reaction in water (**a**) thermal denaturation: heat promotes W_I_ to W_III_. (**b**) Chemical denaturation: denaturant combines with W_II_ (in both processes the equilibrium is displaced toward iceberg formation). (**c**) Stabilizers are good templates for tetrahedral structure of W_I_ (as Implicit Solvent) and the equilibrium is displaced toward iceberg reduction.

A mechanism analogous to that of thermal denaturation (Figure 2a) can explain the chemical denaturation of proteins (Figure 2b). The added denaturant tends to combine with water W_II_ thus displacing the equilibrium in water toward hydrophobic hydration with iceberg formation. The negative entropy produced by iceberg formation neutralises the positive entropy of folding. In other words, we can say that heat displaces the equilibrium of water forms toward hydrophobic hydration by acting on W_III_ whereas the denaturant displaces the equilibrium by acting on W_II_. The negative entropy Δ*S_for_* << 0 that is produced has the same effect as in heat denaturation, destroying the hydrophobic bonds of the native protein. With reference to the equilibrium in water, we can explain [4] the action of the so-called stabilizers, substances that favor protein folding. We can consider (Figure 2c) that the stabilizers act as templates for the tetrahedral structure of water W_I_, thus displacing the equilibrium in the opposite direction, toward the reduction of the iceberg and hydrophilic hydration. 

### 3.2. Motive Free Energy and Iceberg Formation/Reduction

As already explained, the hydrophobic hydration processes are based on the formation of a niche filled with an iceberg in Class A and on iceberg reduction in Class B. The iceberg is formed in Class A, by a phase transition in the bulk of the solvent (W_I_) of +ξ_w_ water clusters W_I_ which then transform into icebergs W_II_. In contrast, the opposite process takes place in Class B whereby an iceberg is reduced in Class B by condensation to solvent (W_I_) of −ξ_w_ water clusters W_I_, reconstituted by molecules W_III_ combined with W_II_ set free by iceberg reduction. The number ξ_w_ depends on the size of the reacting molecule or moiety of macromolecule. As shown in the previous sections, we have accepted the idea that ξ_w_ water clusters W_I_ give origin to a change of phase forming (W_II_ + W_III_) in such a way that the values of enthalpy divided by T, ΔH_th_/T and entropy, ΔS_th_, produced by this change of phase, consist of the only contribution by the hydrophobic isobaric heat capacity ΔC_p,hydr_ in every hydrophobic hydration process. The existence of thermal entropy and thermal enthalpy as distinct from motive entropy and motive enthalpy, respectively, follows from the Dual-Structure Partition Function {DS-PF} [5]. If we apply this principle, by subtracting ΔH_th_ from ΔH_dual_ and ΔS_th_ from ΔS_dual_, thus obtaining ΔH_mot_ and ΔS_mot_, respectively, we can calculate a motive free energy,

ΔG_mot_*=* ΔH _mot_*−* TΔS_mot_ (6)

that in protein denaturation, and in general in Class A, is positive (Δ*G_mot_* > 0), exclusively due to the prominent negative configuration entropy contribution Δ*S_for_* for niche formation. The processes of Class A can be classified as entropy opposed. We remind that the values of Δ*H_mot_* and Δ*S_mot_* are simply 

**Figure 3:**
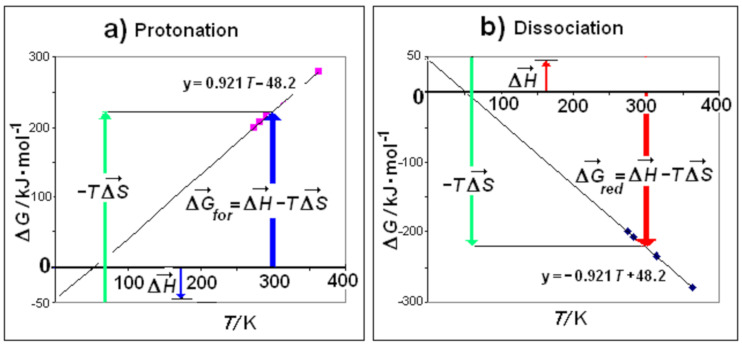
Free energy for (**a**) Class A, with iceberg formation, Δ*G_for_* > 0 (**b**) Class B, with iceberg reduction, Δ*G_red_* < 0. In both Classes, entropy term is predominant. The data in the figure refer to protonation/dissociation of carboxylic acids (*ξ_w_* = 2.1). Dissociation is entropy driven.

Obtained from the experimental data of Δ*H_dual_* and Δ*S_dual_* extrapolated to *T* = 0 and to ln*T* = 0, respectively. This finding is a correction of the opinion of Lumry [6], who retained that motive and thermal parts of enthalpy and entropy were usually not experimentally determinable. The numerical results of the disaggregation of the motive functions for different types of hydrophobic hydration processes are reported in Table 5. Each motive function is composed by two terms. For example, the first term of enthalpy in Class A is Δ*H*_0_^(^*^ξw^*^ = 0)^ and represents the motive enthalpy extrapolated to *ξ_w_* = 0, i.e., at null iceberg. The second term –Δ*h_for_·ξ_w_* represents the contribution to enthalpy by the process of iceberg formation (or iceberg reduction in Class B). Analogous distinctions hold for motive entropy. The reliability of the motive functions, with the terms corresponding to the process of iceberg formation/ reduction, can be checked with reference to the process of protonation of carboxylate anions. This process belongs to Class A with iceberg formation whereas the process of deprotonation of the corresponding acid belong to Class B with iceberg reduction. By considering a reaction of this kind involving *ξ_w_* = 2.1 water molecules W_III_, we can calculate the free energy for iceberg formation as Δ*G_for_* = −*ξ_w_* 22.95 –*T* (−*ξ_w_* 0.4385) = −48.195 + 0.921 *T*. This equation is represented as vector composition in Figure 3a. On the contrary, the free energy for iceberg reduction is represented by the equation Δ*G_red_* = 48.195–0.921 *T* and is represented in Figure 3b. In both diagrams, we can verify how the prominent contribution, either positive or negative, respectively, derives from the entropic component. The process in Class A (Δ*G_for_* > 0) is unstable whereas the process in Class B is thermodynamically favored (Δ*G_red_* < 0). The result is that the dissociated state is the stable one. This corresponds to the experimental finding that carboxylic acids are dissociated in aqueous solution in their stable state.

The strength of each acid is mainly determined by the positive entropic contribution for iceberg reduction. The dissociation of carboxylic acids is entropy driven. We have verified [3] that by subtracting the free energy for iceberg formation (Δ*G°_for_*/*T* = *R*ln*K_for_*) from the free energy for protonation (Δ*G°_prot_*/*T* = *R*ln*K_prot_*) at different temperatures we obtain a residual (Δ*G°_x_*/*T* = *R*ln*K_x_*). The values of ln*K_x_* for every acid plotted against 1/*T* present perfectly linear van’t Hoff plots (i.e., linear binding function). This confirms that was the iceberg reaction to produce the constant curvature of the binding functions.

The analysis of the functions extrapolated to null iceberg Δ*H*_0_^(^*^ξw^*^ = 0)^, Δ*S*_0_^(^*^ξw^*^ = 0)^ and Δ*G*_0_^(^*^ξw^*^ = 0)^(298) offer important pieces of information. The null iceberg functions are referred to the initial step of a reaction: water-gas, water-liquid, lateral chain-lateral chain, etc. In every case, either in Class A or in Class B, the null-iceberg functions, are a small portion of the whole respective motive function. This confirms again that the process of iceberg formation or reduction is ruling the whole reaction. 

The values of free energy Δ*G*_0_^(^*^ξw^*^ = 0)^(298) is positive in Class A and negative in Class B, thus showing which processes are thermodynamically favored with Δ*G*_0_^(^*^ξw^*^ = 0)^(298) < 0. As for enthalpy, the value of Δ*H*_0_^(^*^ξw^*^ = 0)^ = +211.6 kJ·mol^−1^ shows that at uncoiling, the detaching from one another of parts of the coiled external chains of a protein, requires expenditure of energy. 

As for enthalpy, important pieces of information can be extracted from the analysis of Δ*S*_0_^(^*^ξw^*^ = 0)^, at null iceberg. In gas dissolution, the value Δ*S*_0_^(^*^ξw^*^ = 0)^ = −86.4 J K^−1^·mol^−1^ is coincident, with opposite sign, with the entropy change Δ*S_evap_* = +86.9 ± 1.4 J·K^−1^·mol^−1^ (thermal entropy change) given by the Trouton constant referring to the passage in general from liquid to vapour. If we recall that evaporation is the passage from condensed liquid to gaseous vapour we can explain how in the dissolution in aqueous solution, the gas molecule, when trapped in the solvent water, is losing an equivalent amount of configuration entropy. Indirectly, this finding is confirmed by analyzing the process of dissolution in water of liquid substances. In the case of liquids, the entropy change at null iceberg is Δ*S*_0_^(^*^ξw^*^ = 0)^ = −0.5 J·K^−1^·mol^−1^, i.e., almost zero, because the liquid is already condensed before dissolution in water and no condensation process takes place. 

The coincidence of Δ*S*_0_^(^*^ξw^*^ = 0)^ = −86.4 J·K^−1^·mol^−1^, (configuration entropy change) calculated in non-polar gases [3] with the entropy change Δ*S_condens_* = −86.9 ± 1.4 J·K^−1^·mol^−1^ (thermal entropy change) given by the Trouton constant referring to the passage in general from vapour to liquid (equivalence between thermal and configuration entropy) is a highly significant validation of the model. 

The motive functions Δ*H_mot_*, Δ*S_mot_*, and Δ*G_mot_* can be calculated also for micelle formation and for any element of Class B, where the function Δ*G_mot_* results to be negative (Δ*G_mot_* < 0). The process of micelle formation by hydrophobic bonding, notwithstanding the positive enthalpy contribution, is thermodynamically favored (Δ*G_mot_* < 0), because of the overwhelming effect of the favorable entropy contribution (−*T*Δ*S_red_* << 0) for iceberg reduction. The processes of Class B can be classified as entropy driven [5]. 

The analysis of the null iceberg function Δ*S*_0_^(^*^ξw^*^ = 0)^ explains the mistake taken by Chandler [11,12] by introducing the concept of a length effect and of a cross-over point for the passage from volume hydrophobic effect to surface hydrophobic effect. The length scale effect supposed by Chandler is referred to the specific initial state Δ*S*_0_^(^*^ξw^*^ = 0)^ of the reactants, analogous to the initial passage of whole molecules in the dissolution of gases in water (Δ*S*_0_^(^*^ξw^*^ = 0)^ = −86.4 J K^−1^·mol^−1^) or to the dissolution of whole liquid molecules in water (Δ*S*_0_^(^*^ξw^*^ = 0)^ = −0.5 J·K^−1^·mol^−1^) or to the passage from molecular solution to macromolecular solution. In macromolecular solution, the initial state is a free unit with unfolded moieties and the resulting state is that with folded moieties: the macromolecular behavior of folding starts at large macromolecular size. In contrast with Chandler theory, there is no length scale effect for the specific reaction of iceberg formation or iceberg reduction: the kind of iceberg reaction is independent from the size of the solute molecule, rather the affinity of the iceberg reaction is strictly proportional to the size of the entering molecule or moiety, as shown by the constant values of the unitary functions referred to *ξ_w_* = −1 in Table 4, <Δ*h_red_*> = +23.7 ± 0.6 kJ·mol^−1^ ·*ξ_w_*^−1^ and <Δ*s_red_*>_B_ = +432 ± 4 J·K^−1^·mol^−1^·*ξ_w_*^−1^ calculated from any kind of molecule, from noble gases to macromolecules. 

It is worth-noting that the prominent entropic effects, negative in Class A and positive in Class B, respectively, are not consistent with some theories (Ben Naim [8]) attributing hydrophobic or hydrophilic hydration to solute-solvent energetic interactions, that should be stronger or weaker than the solvent–solvent energetic interactions. 

Even the process of cold denaturation of protein can be explained by the *EAM* model [13] by referring to the motive free energy. The change of sign of the motive free energy of folding Δ*G_mot_* (here named Δ*G_fold_*) indicates which is the stable state: either folded or denatured. Above *T_fold_* the folded state is stable being Δ*G_fold_* < 0, whereas below *T_fold_* the protein denatures for Δ*G_fold_* > 0.

### 3.3. Null Thermal Free Energy

Lee and Graziano [14] expressed the opinion that in biochemical processes there are some side reactions where enthalpy and entropy compensate for each other and do not influence the free energy. The same hypothesis had been launched by Benzinger [15]. The Ergodic Algorithmic Model (*EAM*) confirms how thermal enthalpy, Δ*H_th_* and thermal entropy, Δ*S_th_* satisfy the conditions foreseen by these authors. A necessary consequence of this property is that thermal free energy is zero (Δ*G_th_* = 0). Regrettably, thermal free energy is considered different from zero in too many text-books and articles, with specific reference to protein unfolding [16,17,18,19,20] and to micelle formation [21]. Moreover, no mention of the motive functions is reported in these texts [13].

It is worth mentioning that the equation,

Δ*G*(*T*) = 0 − (Δ*C_p,hydr_*/*T*) {(*T_d_* –*T*) + *T* ln(*T*/*T_d_*)} (7)

with Δ*G*(*T*) ≠ 0, represents a heresy for general thermodynamic theory because thermal *intensity entropy* cannot produce any chemical work in a non-reacting system (*NoremE* ensemble), the solvent, wherein no concentration change is possible and, consequently no free energy can be produced. Mechanical work, however, is possible in these non-reacting systems (cf. Carnot cycle). On the other hand, the mathematical expression of Equation (7), if correctly developed, results to be equal to zero, as required by thermal partition functions. Therefore, we obtain, at variance with the erroneous Equation (7):

Δ*G*(*T*)/*T* = Δ*H*(*T*)/*T*–Δ*S*(*T*) = 0 (8)

[5] in accordance to the invariable property of null free energy (Δ*G_th_*/*T* = 0) of thermal functions.

We would like to underline that the identification of the solvent as a non-reacting system (implicit solvent) has been possible because of the introduction of distinct partition functions for Implicit Solvent, with thermal probability factor {*T-PF*}, and for solution with motive probability factor {*M-PF*}. The null free energy is a constitutional invariable property of every non-reacting molecule ensemble (*NoremE*) [5].

### 3.4. Water W_I_, W_II_, W_III_, and Hydrophobic Bond

**Figure 4:**
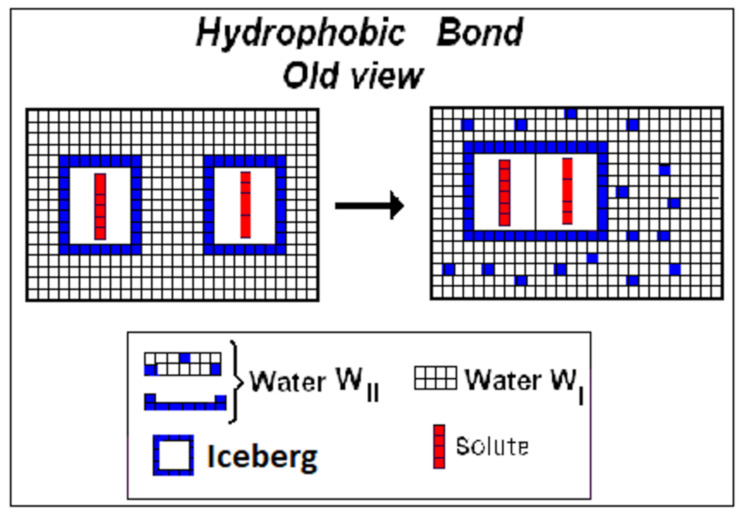
Old view of hydrophobic bonding. The molecules W_III_ expelled from solute–solvent interface are erroneously assumed to increase the configurational entropy of the system. Such a molecular mechanism is expected to express Δ*C_p.hydr_* > 0, contrary to the experimental results.

Characterization of hydrophobic bonding is another point of the thermodynamics of hydrophobic hydration processes where the Ergodic Algorithmic Model (*EAM*) can offer a positive contribution to modify erroneous assumptions, unfortunately accepted by the literature. While examining the applicability of the second law of thermodynamics to the living organisms, Edsall and Gutfreund [22] have considered the assembly of a virus molecule from its subunits, which, according to these authors, apparently involves an increase of order in the system. If the virus is considered an isolated system, this process—according to them—would be in defiance of the Second Law. However, a virus molecule—they arbitrarily assume—interacts directly with its environment. The assembly of a virus molecule was assumed by Edsall and Gutfreund (Figure 4) to increase the entropy of the whole system, due to the supposed liberation of solvation water from the components and the resulting increase in rotational and translational entropy of solvent molecules, when detached from the interface between subunits. The Ergodic Algorithmic Model rejects the generally accepted interpretation (cf. Wikipedia) of “the assembling of a virus molecule from the components with expulsion of solvent molecules from the intermolecular interface, with increase in rotational and translational entropy of the solvent molecules expelled”.

**Figure 5:**
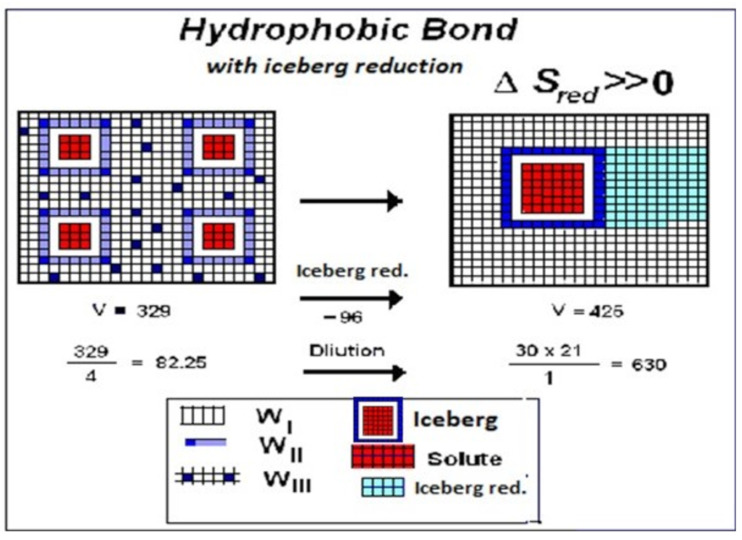
Association of four macromolecular units by hydrophobic bonding, with iceberg reduction. The conventional solvent volume is evaluated from the number of empty and emeraldine squares. Reduction of the iceberg (emeraldine) is accomplished by condensation of water molecules W_III_ and W_II_ as water W_I_ with consequent increase of solvent volume. Contemporarily four solute units become one unique unit and this process also increases dilution. An increase of dilution corresponds to an increase of solute entropy.

The hydrophobic association processes present invariably negative Δ*C_p.hydr_* (Δ*C_p.hydr_* < 0). According to the Ergodic Algorithmic Model (EAM), negative heat capacity means negative *n_w_* and condensation of water molecules to form W_I_. (We recall that Δ*C_p.hydr_* = *n_w_C_p.wr_* and *ξ_w_* = |*n_w_*|). Therefore, association by hydrophobic bonding means that the reaction **B**(−*ξ_w_*W_III_– *ξ_w_*W_II_(iceberg) → *ξ_w_*W_I_ has taken place, with condensation of water molecules W_II_+W_III_ to W_I_ and consequent iceberg reduction. If we would accept the suggestion of Edsall and Gutfreund of the liberation of water of solvation from the interface, we should have Δ*C_p.hydr_* > 0 contrary to the experimental evidence. In any case, we have shown above that the thermal components of the thermodynamic functions cannot give any contribution to free energy (−Δ*G_th_/T* = 0). In contrast, the Ergodic Algorithmic Model (EAM) (Figure 5) suggests that the initially separated units (supposed to be four) stick together with coalescing of the icebergs. The reduction of iceberg by condensation of water molecules W_II_ and W_III_ to W_I_, results in an increase (emeraldine) of the solvent volume. 

The increase of the solvent volume (Δ*V_solvent_* > 0) is combined with the reduction of the number of independent molecular units of the solute from 4 to 1, so that the solute is diluted. Dilution of solute means increase of density entropy. Moreover, water molecules W_III_ disappear from the solution for condensation, thus becoming more diluted as ligand. Altogether, these combined processes make the dilution of the solute to increase.

Correspondingly, Tanford also considers the formation of hydrophobic bonds. According to this author, when two or more hydrophobic molecular units present in the solution associate one another the extension of the solute–solvent interface should be reduced. 

Consequently, the number of hydrogen bonds rearranged should be reduced, thus producing, according to Tanford [23], positive entropy gaining in the solvent water. The last process has some resemblance with the reaction **B**(−*ξ_w_*W_III_–*ξ_w_*W_II_ → *ξ_w_*W_I_). The process of iceberg reduction is completely ignored and the entropy increase erroneously attributed to the solvent but not to the solute. We confirm again that only the implicit solvent is consistent with the convoluted binding potential functions.

According to the Ergodic Algorithmic Model (EAM), the increase of configuration density entropy due to dilution is the driving force that moves the reaction toward the association of the units by hydrophobic bonding. In other words, the entropy change can be attributed exclusively to changes in the thermodynamic state of the solute, {*M-PF*}. and not of the solvent {*T-PF*}.

Alternatively to the scheme of Edsall and Gutfreund, the dissolution of molecules has been interpreted by Tanford [23] as a rearrangement of water-to-water hydrogen bonds. This rearrangement should be caused by the introduction into the solvent water of molecules of a hydrophobic compound, e.g., an aliphatic hydrocarbon. This process should involve entropy-consuming reactions in the solvent. The rearrangements of hydrogen bonds take place at the solute-water interface. This process resembles the reaction **A**(*ξ_w_*W_I_ → *ξ_w_*W_II_ + *ξ_w_*W_III_) without iceberg formation. The step of iceberg formation is again completely ignored by Tanford: the entropy changes is still erroneously attributed to the solvent and not to the solute. The difference between the Edsall and Gutfreund [22] scheme and that proposed by the Ergodic Algorithmic Model (EAM) (Figure 5) is evident. In the Edsall and Gutfreund scheme (see Figure 4), and in that of Tanford as well, the entropy producing processes take place in the structure of the solvent, with changes in the thermodynamic thermal parameters of the solvent itself.

In contrast, in the Ergodic Algorithmic Model (EAM), the entropy consuming process of iceberg formation or the entropy producing process of iceberg reduction taking place in the solvent yield changes in the thermodynamic configuration state of the solute {*M-PF*}. In fact, iceberg formation means diminution of dilution of the solute and, therefore, density entropy diminution, whereas iceberg reduction with extension of the solvent volume is equivalent to increasing the dilution and hence increasing the density entropy of the solute. These entropy changes of the solute are more pertinent to the problem at hand: we are studying, in fact, the thermodynamic properties of the solute.

This new view of hydrophobic bonding represents a complete change of perspective with respect to the Edsall and Gufreund scheme. It is important from a theoretical point of view, to underline this point. The assignment of a change of configuration density entropy to the solvent W_I_ is in principle contradictory. A chemical reaction consists of changes of the concentrations of the reactants, corresponding to changes in configuration density entropy: it is impossible to have a change of concentration (or dilution) of an excess component, the solvent (*NoremE* ensemble, as Implicit Solvent), that has, by definition, no concentration change. The solvent in a diluted solution has the same role of vacuum in a gas. The vacuum can only change its volume but not its concentration and is at constant potential. Therefore, the solvent cannot produce per se any configuration density entropy. In fact, in the Ergodic Algorithmic Model (EAM) that part of water giving origin to increase, or diminution of configuration entropy is water W_II_, or W_III_ as factors of equilibrium constant, whereas the function of solvent (as the implicit solvent) is reserved for water W_I_. Therefore, the idea of considering that the favorable entropy causing hydrophobic bonds is generated within the solvent water is unacceptable.

**Figure 6:**
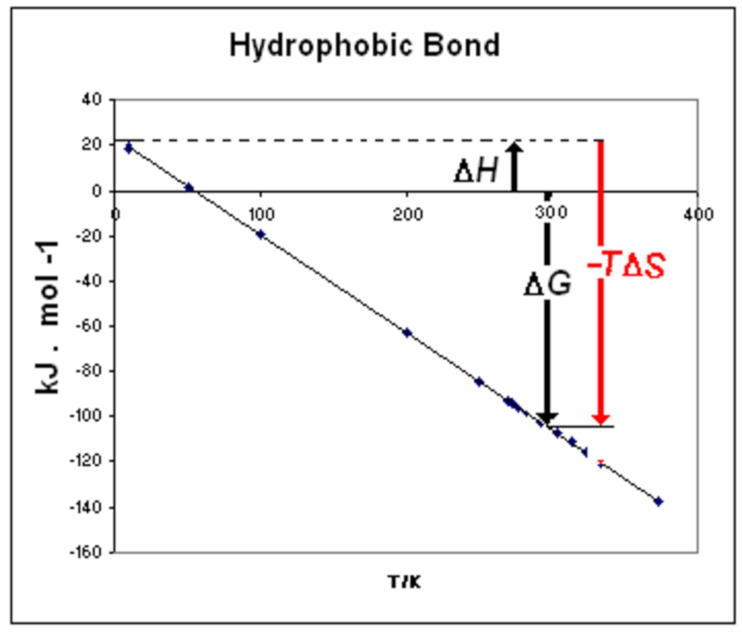
The entropy term is the prominent contributor to the negative free energy (at 298 K) of the hydrophobic bond (entropy-driven).

The free energy change for hydrophobic bonding can be evaluated from the mean values for iceberg reduction, <Δ*h_red_*>_B_ = +23.7 ± 0.6 kJ·mol^−1^·*ξ_w_*^−1^ and <Δ*s_red_*> _B_ = +432 ± 4 J·K^−1^·mol^−1^·*ξ_w_^−^*^1^ reported in Table 4. At 298 K, the free energy for hydrophobic bond results to be Δ*G*_298_ = −105.04 kJ·mol^−1^·*ξ_w_*^−1^ for each water molecule W_III_ involved. The composition of this free energy is represented in Figure 6 where we can appreciate the overwhelming effect of the entropy term. The hydrophobic bond is confirmed to be entropy driven. We repeat, that we are dealing with positive entropy change of the solute, due to iceberg reduction, with consequent expansion of the solvent volume with dilution of the solute.

The separation of the thermal functions Δ*H_th_* and Δ*S_th_* from the motive functions Δ*H_mot_* and Δ*S_mot_*, respectively, raises the question whether the temperature is conditioning, or not, hydrophobic bonding. The separate determination of the thermal functions Δ*H_th_/T* and Δ*S_th_* which are equal to each other leads to conclude that both represent the same entropy change (both are measured in J·K^−1^·mol^−1^). The thermal portions Δ*H_th_* and Δ*S_th_* of the observed thermodynamic functions Δ*H_dual_* and Δ*S_dual_*, respectively, concern the transformation (phase transition) of water W_I_. 

The thermal functions, concerning the solvent partition function, do not contribute to free energy of iceberg reduction or iceberg formation which are the basic steps for formation or disruption, respectively, of the hydrophobic bond. Therefore, this shows that it is vain to search for a direct effect of the temperature on the hydrophobic processes of iceberg formation or reduction. Indirectly, it is the supply of heat at denaturation that promotes [4] melting of some water clusters W_I_ and causes iceberg formation.

### 3.5. Water W_I_: Implicit Solvent

The question of the changes in the volume of the solvent is of some concern for General bio-thermodynamics because it is connected to the type of solution model adopted. In the theory of ideal solution, in fact, the solvent is like a vacuum. The solute molecules move in this surrounding as if they were gaseous. The only transformation undergone by the solvent concerns its volume. According to the Ergodic Algorithmic Model (EAM), the solvent water, in its component W_I_, as Implicit Solvent, keeps the properties of the bulk solvent. Changes in the volume of the solvent produce changes in the thermodynamic properties of the solute. The only changes that are relevant to the thermodynamic properties of the solvent are “iceberg formation” (Δ*V_solvent_* < 0), increasing the concentration of the solute, or “iceberg reduction” (Δ*V_solvent_* > 0) increasing the dilution of the solute. Iceberg formation is entropy consuming (<Δ*s_for_*>_A_ = −445 ± 3 J·K^−1^·mol^−1^·*ξ_w_^−^*^1^), whereas iceberg reduction is entropy producing (<Δ*s_red_*>_B_ = +432 ± 4 J·K^−1^·mol^−1^ ·*ξ_w_*^−1^). We recall that the large entropy production:

(i) is developing in the solute motive partition function {*M-PF*}, and
(ii) is the driving force forming hydrophobic bonds between solute units.

The picture of a gain of entropy by the solute, as due to dilution of the solute itself, is coherent with the so-called molecule-frame (MF) approach. According to Henchman et al. [24] the MF approach is associated with theories such as *“continuum solvent”*. This approach ignores the explicit nature of the solvent using vacuum in the ideal gas as reference model. The MF approach is valid under the condition of diluted solutions, condition that hydrophobic hydration processes satisfy. Alternatively, Henchman proposes to refer to the system frame (SF) whereby the molecules, either of solute and solvent, are referred to a unique common reference system. The MF frame seems more adequate for the ergodic algorithmic model, whereby water W_I_ represents the Implicit Solvent with thermal partition function {*T-PF*}. In fact, the cluster W_II_ surrounding the solute forms a unique molecular unit with a solute moiety and it follows the thermodynamic state of that solute moiety, in the realm of {*M-PF*}. The (solute +W_II_) molecular unit is dissolved in the bulk solvent W_I_, again conform to the *MF* scheme. At the same time, the water molecules W_III_ are free to move in the full volume of the solvent W_I_, as in a vacuum, and this part of the process is again conformed to the *MF* picture.

The processes of iceberg reduction and iceberg formation imply enthalpy changes also. The enthalpy for iceberg formation indicates an exothermic reaction **A**(*ξ_w_*W_I_ → *ξ_w_*W_II_(iceberg) + *ξ_w_*W_III_ (<Δ*h_for_*>_A_ = −22.2 ± 0.7 kJ·mol^−1^·*ξ_w_*^−1^). This is the unitary enthalpy change for the transformation from clusters (W_x_)_I_ to ((W_x-1_)_II_ + W_III_) with iceberg formation and chemical combination with solute to form the solvation sheath. This reaction producing the ligands of the solute (W_x-1_)_II_ and W_III_ with solute solvation takes place in the domain of motive partition function and is of concern, therefore, for the motive thermodynamic functions. The transformation from clusters (W_x_)_I_ to clusters (W_x-1_)_II_ implies changes in the strength of water-water hydrogen bonds that are stronger in W_II_ than in W_I_. The component W_II_ is credited, in fact, of higher density than W_I_. The reaction step of iceberg formation with solvation of the solute is, therefore, exothermic. On the other hand, the process of iceberg reduction **B**(−*ξ_w_*W_III_ − *ξ_w_*W_II_(iceberg) → *ξ_w_*W_I_), that involves a back reaction from ((W_x-1_)_II_+W_III_) to (W_x_)_I_, is endothermic as shown by the unitary enthalpy <Δ*h_red_*>_B_ = +23.7 ± 0.6 kJ·mol^−1^·*ξ_w_*^−1^. The enthalpy changes are again coherent with the *MF* approach because the enthalpy effects concern only water clusters W_II_ and molecules W_III_ solvating the solute (i.e., all solute components), whereas clusters (W_x_)_I_, composing the bulk solvent, do not change their concentration but simply expand or reduce their total volume by addition or subtraction, respectively, of some clusters.

### 3.6. From Ergodic Algorithmic Model to Computer Chemistry

In Reference [13], we have already discussed the connections between Ergodic Algorithmic Model (EAM) and computo-chemistry, particularly between EAM and Potential Distribution Theorem (PDT). The comparison has revealed the relevant weak points of PDT. Many calculations aiming at obtaining potential functions *μ_s_* for iceberg formation in the solvent, based on the partition function of the solvent itself, are not appropriate because, as shown by the Ergodic Algorithmic Model (EAM), the thermal partition function {*T-PF*} of the solvent cannot give origin to any free energy change and, of course, to any change of potential *μ_s_*. In contrast, another point of *PDT* has been properly developed, in conformity with the dual structure of the hydrophobic hydration systems. The introduction in *PD* by Pratt and La Violette of the *quasi*-chemical approximation [25,26], which could be more appropriately renamed as *chemical molecule/mole scaling function,* keeps the point that the solute of any hydrophobic hydration system constitutes a *REME* ensemble, not ruled by Boltzmann statistics, rather by binomial distribution of chemical reactions. The partition function of the solute is the Motive Partition Function {*M-PF*} and the binding function *RT*ln*K_mot_* = *f*(*T*) can be calculated by applying the *quasi*-chemical approximation, proposed by Pratt and La Violette [25,26] either to the dissociation constant of water

*K_diss_ =* (*a*_A_)·(*a*_B_)^−1^·(*a*_W__II_)^ξ^*^w^* (9)

or to the association constant of water

*K_assoc_ =* (*a*_A_)·(*a*_B_)^−1^·(*a*_W_II__)^–^*^ξ^^w^* (10)

chosen by reference to the sign of ±*ξ_w_*, experimentally determined. The *ergodic* activity *a*_A_ is calculated as product of thermal activity factor *Φ* times molar fraction *x*_A_

*a*_A_* = Φ*·*x*_A_ (11)

where *Φ* = *T* ^–(*C*^*^p,^*^A */R*)^ is preserving the ergodic property of the solution. By taking advantage of the previous calculation of the thermal functions Δ*H_th_* = Δ*C_p,hydr_T* and Δ*S_th_* = Δ*C_p,hydr_* ln*T*, the calculated binding potential functions α) ln*K_calc_* = (−Δ*G_calc_*/*T*) = {*f*(1/*T*)_*_g(*T*)}, and β) *RT*ln*K_calc_* = (−Δ*G_calc_*) = {*f*(*T*)_*_g(*lnT*)} can be obtained and numerically compared with the observed binding potential function *R*ln*K_dual_* = (−Δ*G_dual_*/*T*) = {*f*(1/*T*)_*_g(*T*)} and *RT*ln*K_dual_* = (−Δ*G_dual_*) = {*f*(*T*)_*_g(*lnT*)}.

Unfortunately, too many computer simulations are not recognizing the dual structure of the partition function {*DS-PF*} of the hydrophobic hydration processes, and do not combine the binomial distribution of a Mole ensemble {*M-PF*} with the Boltzmann distribution of a molecule ensemble {*T-PF*}. The computer calculations should be amended, by following the procedure indicated by Talhout, et al. [27], who have determined experimentally the curved binding functions at different temperatures for a series of hydrophobically modified benzamidinium chloride inhibitors to trypsin, and then have checked the results of simulations with the experimental findings.

We recall the point that the statistical validation of the whole set of experimental data of a significant large population of experimental points relative to hydrophobic hydration processes of any kind, presented in this article, qualifies these data as representative of any type of hydrophobic hydration processes. These results realize the *user-friendly functions* hoped for by Lumry. Therefore, in every example of computer-assisted drug design, we must assume that binding potential functions α) *RT*ln*K_dual_* = (−Δ*G_dual_*/*RT*) = {*f*(*T*)_*_g(*lnT*)**}** and and β) *R*ln*K_dual_* = (−Δ*G_dual_*) = *f*(1/*T*)_*_(*T*) *necessarily* exist. These functions can be experimentally determined in advance of computer simulation. Then, computer simulations will be assessed in comparison with experimental equilibrium constants.

## 4. Conclusions

The hydrophobic hydration processes are characterized, from a thermodynamic point of view by curvilinear shapes of the binding potential functions α) *R*ln*K_dual_* = (−Δ*G_dual_*/*RT*) = {*f*(1/*T*)*g(*T*)} and β) *RT*ln*K_dual_* = (−Δ*G_dual_*) = {*f*(*T*)*g(*lnT*)}. The Class A processes present each function (convex), with a minimum whereas the Class B processes present each function (concave), with a maximum. The type of curvature depends on the type of reaction in water. In Class A, the reaction of water with phase transition (solvent → iceberg) is

**A**{ξ*_w_*W_I_(solvent) → ξ*_w_*W_II_(iceberg) + ξ*_w_*W_III_}

whereas in Class B, the reaction of water with opposite phase transition (iceberg → solvent) is

**B**{– ξ*_w_*W_III_– ξ*_w_*W_II_(iceberg)→ ξ*_w_*W_I_(solvent)}

The curvatures of the binding functions depend on the non-zero value of the hydrophobic heat capacity Δ*C_p.hydr_* (Δ*C_p.hydr_* ≠ 0). The hydrophobic heat capacity Δ*C_p.hydr_* (Δ*C_p.hydr_* = ±*ξ_w_C_p.w_*) is constant and independent from the temperature because it depends on the number ±*ξ_w_* of water molecules W_III_ involved in each specific hydrophobic hydration process. Being ±*ξ_w_* a *pseudo*-stoichiometric coefficient, it must remain necessarily constant if the reaction remains the same at different temperatures. The determination, therefore, of the curvatures of the binding functions α) *R*ln*K_app_* = (−Δ*G_dual_*/*T*) = {*f*(1/*T*)_*_g(*T*)} and β) *RT*ln*K_dual_* = (−Δ*G_dual_*) = {*f*(*T*)_*_g(ln*T*)}, respectively, represents a new reliable and efficient method, based on *TED,* for measuring the number ±*ξ_w_* of water clusters W_I_ and hence of water icebergs W_II_, and water molecules W_III_.

The observed dual enthalpy Δ*H_dual_* and the observed dual entropy Δ*S_dual_* are composed each by two terms, thermal and motive: Δ*H_dual_* = Δ*H_mot_* + Δ*H_th_* and Δ*S_dual_* = Δ*S_mot_* + Δ*S_th_*, respectively. A typical property of Δ*C_p.hydr_* is that it contributes exclusively, and is the only contribution, to the thermal components of the thermodynamic functions, Δ*H_th_* and Δ*S_th_*. These thermal functions concerning the solvent W_I_ only, represent the thermal entropy acquired (Δ*H_th_*/*T* = +*ξ_w_C_p.w_*) in Class A by those water molecules W_I_ that, by a phase change, become water W_II_ with W_III_. In Class B, the thermal functions represent thermal entropy lost (Δ*H_th_*/*T* = −*ξ_w_C_p.w_*) by those water molecules W_II_ with W_III_ that go back to water W_I_. The thermal components give a null contribution to free energy (−Δ*G_th_/T* = 0), although they significantly affect the observed enthalpy and entropy values. The compensative properties follow from the thermal probability factor {*T-PF*}, referred to the solvent.

The motive functions Δ*H_mot_* and Δ*S_mot_* are obtained by subtracting the contributions of the thermal functions from the observed enthalpy, Δ*H_dual_* and entropy, Δ*S_dual_*, respectively. The motive functions, which derive from the motive probability factor {*M-PF*}, are independent from *T* but depend on the stoichiometry ±*ξ_w_* of water clusters W_I_ (solvent) to water W_II_ (iceberg)). The motive functions of each compound in a homogeneous series, disaggregated by plotting them as the function of the respective number *ξ_w_*, give self-consistent unitary values of enthalpy and entropy, in Class A

<Δ*h_for_*>_A_ = −22.7 ± 0.7; kJ·mol^−1^ ·*ξ_w_*^−1^; <Δ*s_for_*>_A_ = −445 ± 3; J·K^−1^·mol^−1^·*ξ_w_*^−1^

and in Class B

<Δ*h_red_*>_B_ = +23.7 ± 0.6; kJ·mol^−1^ ·*ξ_w_*^−1^; <Δ*s_red_*> _B_ = +432 ± 4; J·K^−1^·mol^−1^·*ξ_w_*^−1^

These unitary values present low variability, in the limits of experimental error, notwithstanding they were obtained from data concerning molecules of different size, in different aggregation states and measured by different experimental methods. This means that the about 600 experimental data from about 80 different compounds give origin to a normal population of experimental errors. The statistical inference confirms that Δ*C_p.hydr_* is constant and that the unitary functions calculated are user-friendly to calculate the motive functions in every biochemical equilibrium.

The motive functions concern the partition function {*M-PF*} of the solute. The solute includes water molecules W_III_, as free ligand, and clusters W_II_, as iceberg sheaths joined to other solute units. The motive functions, combined in a Gibbs equation, give the free energy change Δ*G_mot_* concerning the solute in every hydrophobic hydration process. Recognition of the peculiarities of thermal and motive functions is essential for a correct analysis of the thermodynamics of many biochemical equilibria. It is worth note the essential role played by the reaction steps of iceberg formation from water W_I_ (Class A) or iceberg reduction to water W_I_ (Class B) in regard of the motive configuration density entropy of the solute (and not of the solvent) in every hydrophobic hydration process. The motive configuration density entropy change of the solute for iceberg formation is negative in Class A by reducing the volume of the solvent water W_I_ and positive for iceberg reduction in Class B by expanding the volume of the solvent water W_I_. In both Classes, the changes of iceberg have effect on the motive partition function {*M-PF*} of the solute.

The processes of iceberg formation or iceberg reduction are ubiquitous in bio-fluids. The knowledge of the user-friendly unitary functions reported above, coupled to Thermal Equivalent Dilution (TED) method for the determination of the number ξ*_w_*, will be of fundamental help for anybody interested in the studies of the biochemical equilibria. By employing the user-friendly unitary functions, in the future, anybody can calculate the motive functions (enthalpy, entropy, and free energy) for iceberg reaction in any compound, if one has previously determined, by applying TED, the coefficient *ξ_w_* for each compound. Even computer simulations can take advantage of the information provided by Ergodic Algorithmic Model (EAM) to check the reliability of the numbers obtained by statistical mechanics calculations. The *quasi*-chemical approximation [25,26] can be employed to feel the gap between *NoremE* and *REME* ensembles.

The validity of the iceberg model in the literature is controversial. Indeed, the results obtained from experiments and calculations carried out to prove the usual iceberg model are conflicting: the first time-resolved observations concluded that some water molecules are immobilized by hydrophobic groups [28], in strong contrast to previous NMR conclusions [29]. Molecular dynamics simulations of aqueous solutions of various hydrophobic solutes, for a wide range of concentrations, show that the rate of water reorientation in the vicinity of the hydrophobic solutes is decreased only moderately [30]. Our model, even if assumes the iceberg formation, has as a focal point the existence of implicit solvent and a completely different approach to the hydrophobic process. In fact, the positive density entropy (configuration) gain Δ*S_red_* >> 0 for iceberg reduction to water W_I_ with consequent expansion of solvent volume, is the driving force that causes the formation of the hydrophobic bonds. This reappraisal of the hydrophobic bond represents a complete change of perspective with respect to the mechanism proposed in the literature. The role of water is completely reversed: reduction of iceberg W_II_ associated to W_III_ with condensation as W_I_, with consequent density entropy gain by the more diluted solute. No more dissociation of W_II_ erroneously is considered as density entropy increase of the solvent.

## Figures and Tables

**Table 1:** Disaggregation of motive functions for non-polar gases (ξ_w_ = |n_w_|). Data from E. Wilhelm, R. Battino, R.J. Wilcock, *Chem. Rev.*, 1977, *77*, 219–262.

Motive Function	Equation	R^2^ Factor	At Null Iceberg (*ξ_w_* = 0)	St. dev.
Δ*H_mot_* =	+13.124 − 31687 *n_w_*	0.983	Δ*H*_0_^(^*^ξ^^w^*^ = 0)^ = −31.7 kJ·mol^−1^	2.1
Δ*S_mot_* =	−82.681 − 445.44 *n_w_*	0.993	Δ*S*_0_^(^*^ξ^^w^*^ = 0)^ = −82.7 J·K^− 1^·mol^−1^	5.4

**Table 2:** General paradigm for hydrophobic hydration processes.

**Transformations**			
Class A	**A**(*ξ*_w_W_I_ → *ξ*_w_W_II_(iceberg) + *ξ_w_*W_III_)		iceberg formation
Class B	**B**(−*ξ*_w_W_III_ − *ξ*_w_W_II_ (iceberg) → *ξ_w_*W_I_)		iceberg reduction
**Thermodynamic Functions**			
**Class A**			
**Apparent**	**Motive**	**Iceberg**	**Thermal**
Δ*H_dual_* = Δ*H*_0_^(^*^ξ^^w^*^ = 0)^ + Δ*H_for_* + Δ*H_th_*	Δ*H_mot_* = Δ*H*_0_^(^*^ξ^^w^*^ = 0)^ + Δ*H_for_*	Δ*H_for_* = *ξ_w_*·Δ*h_for_* < 0	Δ*H_th_* = +*ξ_w_ C_p,w_* *T*
Δ*S_dual_* = Δ*S*_0_^(^*^ξ^^w^*^ = 0)^ + Δ*S_for_* + Δ*S_th_*	Δ*s_mot_* = Δ*S*_0_^(^*^ξ^^w^*^ = 0)^ + Δ*S_for_*	Δ*S_for_* = *ξ_w_*·Δ*s_for_* < 0	Δ*S_th_* = +*ξ_w_ C_p,w_* ln*T*
**Class B**			
**Apparent**	**Motive**	**Iceberg**	**Thermal**
Δ*H_dual_* = Δ*H*_0_^(^*^ξ^^w^*^ = 0)^ + Δ*H_red_* + Δ*H_th_*	Δ*H_mot_* = Δ*H*_0_^(^*^ξ^^w^*^ = 0)^ + Δ*H_red_*	Δ*H_red_* = *ξ_w_*·Δ*h_red_* > 0	Δ*H_th_* = −*ξ_w_ C_p,w_* *T*
Δ*S_dual_* = Δ*S*_0_^(^*^ξ^^w^*^ = 0)^ + Δ*S_red_* + Δ*S_th_*	Δ*S_mot_* = Δ*S*_0_^(^*^ξ^^w^*^ = 0)^ + Δ*S_red_*	Δ*S_red_* = *ξ_w_*·Δ*s_red_* > 0	Δ*S_th_* = −*ξ_w_ C_p,w_* ln*T*

**Table 3:** Unitary functions by ergodic algorithmic model.

Class A. Unitary Enthalpy Function: Δ*h_for_*			
Process	Δ*h_for_*	Unit	ξ*_w_* range
Gas dissolut.	−21.6	kJ·mol^−1^·*ξ_w_*^−1^	2–6
Liquid dissol.	−23.3	kJ·mol^−1^·*ξ_w_*^−1^	2.7–5.4
Protein denat.	−22.1	kJ·mol^−1^·*ξ_w_*^−1^	80–140
Carbox Proton.(*)	−21.8	kJ·mol^−1^·*ξ_w_*^−1^	1.8–2.3
Class A. Unitary Entropy Function: Δ*s_for_*			
Process	Δs*_for_*	Unit	ξ*_w_* range
Gas dissolut.	−445.4	J·K^−1^mol^−1^·*ξ_w_*^−1^	2–6
Gas (Privalov)	−450	J·K^−1^mol^−1^·*ξ_w_*^−1^	2–6
Liquid dissol.	−447	J·K^−1^mol^−1^·*ξ_w_*^−1^	2.7–5.4
Protein denat.	−428.5	J·K^−1^mol^−1^·*ξ_w_*^−1^	80–140
Carbox Proton.(*)	−442.6	J·K^−1^mol^−1^·*ξ_w_*^−1^	1.8–2.3
Class B. Unitary Enthalpy Function: Δ*h_red_*			
Process	Δ*h_red_*	Unit	ξ*_w_* range
Micelle	+23.2	kJ·mol^−1^·*ξ_w_*^−1^	4–19
Bio-complx	+24.3	kJ·mol^−1^·*ξ_w_*^−1^	19–189
Benz.Cl-tryps.	+23.41	kJ·mol^−1^·*ξ_w_*^−1^	5.3–11.3
Class B. Unitary Entropy Function: Δ*s_red_*			
Process	Δ*h_red_*	Unit	ξ*_w_* range
Micelle	−428±33	J·K^−1^mol^−1^·*ξ_w_*^−1^	4–19
Bio-complx	−436.2	J·K^−1^mol^−1^·*ξ_w_*^−1^	19–189
Benz.Cl-tryps.	−434.4	J·K^−1^mol^−1^·*ξ_w_*^−1^	5.3–11.3

(*)Carboxylic acids.

**Table 4:** Validation of the model. Analysis of unitary thermodynamic functions (*) [3].

Analysis within Classes		
Class A: iceberg formation	Unit	Relative error
<Δ*h_for_*>_A_ = −22.7 ± 0.7	kJ·mol^−1^ *·ξ_w_*^−1^	±3.1%
<Δ*s_for_*>_A_ = −445 ± 3	J·K^−1^·mol^−1^·*ξ_w_*^−1^	±0.7%
Class B: iceberg reduction	Unit	Relative error
<Δ*h_red_*>_B_ = +23.7 ± 0.6	kJ·mol^−1^ *·ξ_w_*^−1^	±2.51%
<Δ*s_red_*> _B_ = +432 ± 4	J·K^−1^·mol^−1^·*ξ_w_*^−1^	±0.9%
Comparison among Classes		
Enthalpy	Entropy	
<Δ*h_for_*>_A_ = −22.7 ± 0.7 kJ·mol^−1^·*ξ_w_*^−1^	<Δ*s_for_*> _A_ = −445 ± 3 J·K^−1^·mol^−1^·*ξ_w_*^−1^	
<Δ*h_red_*>_B_ = +23.7 ± 0.6 kJ·mol^−1^·*ξ_w_*^−1^	<Δ*s_red_*> _B_ = +432 ± 4 J·K^−1^·mol^−1^·*ξ_w_*^−1^	
mean abs.value <Δ*h*>_A,B_ = 22.95 ± 0.75	mean abs.value <Δ*s*> _A,B_ = 438.5 ± 6.5	
mean sd: (0.7^2^ + 0.6^2^) ^1/2^ = 0.92	mean sd: (3^2^ + 4^2^) ^1/2^ = 5	
Student’s ratio: 0.75/0.92 = 0.815	Student’s ratio: 6.5/5 = 1.3	
**Hypothesis**: absolute values in Class A and B are equal: hypothesis accepted (Mean in Class A = mean in Class B with sign reversed)		

(*) Mean values obtained from more than eighty different sets, with about 600 data points. Note the small variability ±σ, indicating that all the points belong to a unique homogeneous statistical population.

**Table 5:** Motive function disaggregation (*).

Process	Motive Function	Disaggregation Equation	Δ*G_mot_*(298)	Unit	*ξ_w_* Range	<*ξ_w_*> Mean
**Class A**						
Gas Dissolut.	Δ*H_mot_*	−17.7 − 21.6 *ξ_w_*		kJ·mol^−1^		
	Δ*S_mot_*	−86.4(!) − 445.4*ξ_w_*		J·K^−1^mol^−1^		
	Δ*G_mot_*(298)	8.04(**) + 111.133*ξ_w_*	+452.4	kJ·mol^−1^	2–6	4
Liq. Dissolut.	Δ*H_mot_*	+4.6 − 23.3 *ξ_w_*		kJ·mol^−1^		
	Δ*S_mot_*	−0.5(!!) − 447 *ξ_w_*		J·K^−1^mol^−1^		
	Δ*G_mot_*(298)	4.74 (**) + 109.9 *ξ_w_*	+437.9	kJ·mol^−1^	2.7–5.4	4
Protein denat.	Δ*H_mot_*	+211.82 − 22.5 *ξ_w_*		kJ·mol^−1^		
	Δ*S_mot_*	+415 − 428.5 *ξ_w_*		J·K^−1^mol^−1^		
	Δ*G_mot_*(298)	−88.15(**) + 105.04ξ*_w_*	+8319	kJ·mol^−1^	80–140	120
Protonation	Δ*H_mot_*	+0.1 − 21.8 *ξ_w_*		kJ·mol^−1^		
	Δ*S_mot_*	+104 − 442.6 *ξ_w_*		J K^−1^·mol^−1^		
	Δ*G_mot_*(298)	−30.9(**) + 104.6*ξ_w_*	+1783	kJ·mol^−1^	1.8–2.3	2.1
**Class B**						
Protein fold.	Δ*H_mot_*	−211.82 + 22.5*ξ_w_*		kJ·mol^−1^		
	Δ*S_mot_*	−415.8 + 424.2*ξ_w_*		J K^−1^·mol^−1^		
	Δ*G_mot_*(298)	+88.15 − 103.9*ξ_w_*	−8281	kJ·mol^−1^	80–140	120
Micelle form.	Δ*H_mot_*	−3.97 + 23.13 *ξ_w_*		kJ·mol^−1^		
	Δ*S_mot_*	+10.2 + 428 *ξ_w_*		J K^−1^·mol^−1^		
	Δ*G_mot_*(298)	–7.01(**) − 104.4*ξ_w_*	–1569	kJ·mol^−1^	4–19	15
Deprotonat.	Δ*H_mot_*	–0.1 − 21.8*ξ_w_*		kJ·mol^−1^		
	Δ*S_mot_*	−104 + 442.6*ξ_w_*		J K^−1^·mol^−1^		
	Δ*G_mot_*(298)	+30.9(**) − 104.6*ξ_w_*	−1783	kJ·mol^−1^	1.8–2.3	2.1

(*)Δ*H*_0_^(^*^ξw^*^ = 0)^ and Δ*S*_0_^(^*^ξw^*^ = 0)^ are the motive functions extrapolated to null iceberg (*ξ_w_* = 0); (**)Δ*G*_0_^(^*^ξw^*^ = 0)^ (*T)* = Δ*H*_0_^(^*^ξw^*^ = 0)^ − *T* Δ*S*_0_^(^*^ξw^*^ = 0)^; (!) configuration entropy loss at gas condensation; (!!) in liquids: no condensation = no entropy loss.

## Data Availability

Data processed are available in the specified literature references.

## List of Symbols

PDT = Potential Distribution Theorem
EAM = Ergodic Algorithmic Model
{*DS-PF*} = Dual Structure Partition Function
{*M-PF*} = Motive Partition Function (Solute)
{*T-PF*} = Thermal Partition Function (Solvent)
*K_dual_* = experimental equilibrium constant
*K_dual_* = ζ*_th_* · *K_mot_* = product partition function
*K_mot_* = motive equilibrium constant (solute, density entropy, *REME* ensemble,)
ζ*_th_* = 1 = thermal partition function (solvent, intensity entropy, *NoremE* ensemble)
DMSGN = dimethionine derivative of chymotrypsinogen A
*C**_p,w_* = 75.36 J·K^−1^mol^−1^ = molar heat capacity for liquid water
Δ*s**_p,w_* = Δ*h**_p,w_*/*T* = *C**_p,w_* = 75.36 J·K^−1^mol^−1^ entropy change for W_I_ → W_II_ + W_III_ in pure water
Δ*C_p,hydr_* = heat capacity in hydrophobic hydration processes
Class A = hydr. hydration process with reaction **A**(*ξ**_w_*W_I_ → *ξ**_w_*W_II_(iceberg) + *ξ**_w_*W_III_
Class B = hydr. hydration process with reaction **B**(−*ξ**_w_*W_III_ − ξ*_w_*W_II_(iceberg) → *ξ**_w_*W_I_
W_I_ (solvent), W_II_ (iceberg), and W_III_ = types of water
*n_w_* = number of water molecules W_III_ in a hydrophobic hydration process (*ξ_w_* = |*n_w_*|)
±*ξ*_w_ = (*ξ_w_* = |*n_w_*|) = ±absolute *pseudo*-stoichiometric number of water molecules W_III_
TED = thermal equivalent dilution principle (−*R*dln{X}*^n^* = *n C**_p,X_* d(ln*T*)
Δ*H_dual_* = experimental enthalpy (Δ*H_dual_* = Δ*H_mot_* + Δ*H_th_* = Δ*H*_0_^(^*^ξ^**^w^*^ = 0)^ + Δ*H_for_* + *ξ_w_ C**_p,w_T*)
Δ*S_dual_* = experimental entropy (Δ*S_dual_* = Δ*S_mot_* + Δ*S_th_* = Δ*S*_0_^(^*^ξ^**^w^*^ = 0)^ *+* Δ*S_for_* + *ξ_w_ C**_p.w_*ln*T*)
Δ*H_th_* = thermal enthalpy (Δ*H_th_* = Δ*C_p,hydr_*·*T*) (in Class A: Δ*H_th_* > 0, in Class B: Δ*H_th_* < 0)
Δ*S_th_* = thermal entropy (Δ*S_th_* = Δ*C_p,hydr_*·ln *T*). (in Class A: Δ*S_th_* > 0, in Class B: Δ*S_th_* < 0)
Δ*G_th_* = thermal free energy (−Δ*G_th_*/*T* = 0)
Δ*H*_0_ = experimental enthalpy from Δ*H_app_* extrapolated to *T* = 0
Δ*H_mot_* ≡ Δ*H*_0_ = motive enthalpy:
in Class A: Δ*H**_mot_* = Δ*H*_0_^(^*^ξ^**^w^*^ = 0)^ + Δ*H_for_*
in Class B: Δ*H**_mot_* = Δ*H*_0_^(^*^ξ^**^w^*^ = 0)^ + Δ*H_red_*
Δ*H_for_* = *ξ**_w_*·Δ*h_for_* < 0 = enthalpy change for iceberg formation (Class A)
Δ*h_for_*>_A_ = −22.2 ± 0.7 kJ·mol^–^^1^·ξ*_w_*^–^^1^ mean unitary (for ξ*_w_* = 1) enthalpy chg. For iceberg formation
Δ*H_red_* = enthalpy change for iceberg reduction (Class B)
<Δ*h_for_*>_B_ = +22.2 ± 0.7 kJ·mol^–^^1^·*ξ**_w_*^–^^1^ mean unitary (for *ξ**_w_* = 1) enthalpy change for iceberg reduction
Δ*S*_0_ = experimental entropy from Δ*S_dual_* extrapolated to ln*T* = 0
Δ*S_mot_* ≡ Δ*S*_0_ = motive entropy:
in Class A: Δ*S**_mot_* = Δ*S*_0_^(^*^ξ^**^w^*^ = 0)^ + Δ*S_for_*
in Class B: Δ*S**_mot_* = Δ*S*_0_^(^*^ξ^**^w^*^ = 0)^ + Δ*S_red_*
Δ*S_for_* = *ξ**_w_* Δ*s_for_*< 0 = entropy change for iceberg formation (Class A)
<Δ*s_for_*> _A_ = −445 ± 3 J·K^−1^·mol^−1^·*ξ**_w_*^−1^, mean unitary (for *ξ**_w_* = 1) entropy change for iceberg formation
Δ*S_red_* = *ξ**_w_* Δ*s_red_* > 0 = entropy change for iceberg reduction (Class B)
<Δ*s_red_*> _B_ = +445 ± 3 J·K^−1^·mol^−1^·*ξ**_w_*^−1^. mean unitary (for *ξ**_w_* = 1) entropy change for iceberg reduction
Δ*G_mot_* = motive free energy (Δ*G_mot_* = Δ*H_mot_* –*T*Δ*S_mot_* ≡ Δ*H*_0_–*T*Δ*S*_0_)
–Δ*G°*/*RT* = ln *K*
*R* ln *K_dual_* = *f*(1/*T*)**g*(*T*), (α) observed convoluted Binding Potential Function in entropy unit
*RT*ln*K_dual_* = *f*(*T*)**g*(*lnT*), (β) observed convoluted Binding Potential Function in enthalpy unit
Δ*H**_j_* = difference between enthalpy levels in reacting *REME* Ensemble
Δ*V_solvent_* = change of solvent volume
*V*_W__I_ = volume of one unit of water cluster W_I_
Δ*V_solvent_* > 0 = −*V_cav_* = change of solvent volume (*V_cav_* = −*ξ**_w_V*_W__I_ = iceberg reduction)
*R* = 8.31451 J·K^−1^· mol^−1^, gas constant
Δ*H*_0_^(^*^ξ^**^w^*^ = 0)^ = Δ*H_for_*extrapolated to null iceberg
Δ*S*_0_^(^*^ξ^**^w^*^ = 0)^ = Δ*S_for_*extrapolated to null iceberg
*ξ**_w_*^–^^1^ = unitary function, for *ξ**_w_* = 1
*T_d_* = denaturation temperature
*T_H_* = temperature at Δ*H_app_* = 0 (*T_min_* in Class A or *T_max_*in Class B)
*T_S_* = temperature at Δ*S_app_* = 0 (*T_min_* in Class A or *T_max_*in Class B)
NoremE = Non-reacting molecule Ensemble (small m = molecule)
REME = Reacting Mole Ensemble (capital M = Mole)
*niche* = portion of water W_I_ losing rigidity to form water W_II_, free water W_III_ and iceberg
